# Alternative mRNA Splicing Generates Two Distinct ADAM12 Prodomain Variants

**DOI:** 10.1371/journal.pone.0075730

**Published:** 2013-10-07

**Authors:** Sara Duhachek-Muggy, Hui Li, Yue Qi, Anna Zolkiewska

**Affiliations:** Department of Biochemistry and Molecular Biophysics, Kansas State University, Manhattan, Kansas, United States of America; International Centre for Genetic Engineering and Biotechnology, Italy

## Abstract

Human *ADAM12*, transcript variant 1 (later on referred to as Var-1b), present in publicly available databases contains the sequence 5′-GTAATTCTG-3′ at the nucleotide positions 340–348 of the coding region, at the 3′ end of exon 4. The translation product of this variant, ADAM12-Lb, includes the three amino acid motif ^114^VIL^116^ in the prodomain. This motif is not conserved in ADAM12 from different species and is not present in other human ADAMs. Currently, it is not clear whether a shorter variant, Var-1a, encoding the protein version without the ^114^VIL^116^ motif, ADAM12-La, is expressed in human. In this work, we have established that human mammary epithelial cells and breast cancer cells express both Var-1a and Var-1b transcripts. Importantly, the proteolytic processing and intracellular trafficking of the corresponding ADAM12-La and ADAM12-Lb proteins are different. While ADAM12-La is cleaved and trafficked to the cell surface in a manner similar to ADAM12 in other species, ADAM12-Lb is retained in the ER and is not proteolytically processed. Furthermore, the relative abundance of ADAM12-La and ADAM12-Lb proteins detected in several breast cancer cell lines varies significantly. We conclude that the canonical form of transmembrane ADAM12 is represented by Var-1a/ADAM12-La, rather than Var-1b/ADAM12-Lb currently featured in major sequence databases.

## Introduction

The Disintegrin and Metalloprotease (ADAM) proteins belong to the M12B adamalysin protease subfamily (http://merops.sanger.ac.uk/, Ref. [1]). Canonical ADAMs are comprised of a prodomain, a metalloprotease domain, a disintegrin domain, a cysteine-rich domain, an epidermal growth factor-like domain, a transmembrane helix, and a cytoplasmic tail. The human genome contains 21 different *ADAM* genes; however, only 13 of these genes encode functional proteases [2], [3]. The catalytically active ADAMs contain the HEXXHXXGXXH motif in their metalloprotease domain, with three zinc-binding histidine residues and a catalytic glutamic acid [4]. The proteolytic activity of the metalloprotease is inhibited by the prodomain. The mechanism of inhibition typically involves a cysteine-switch mechanism, in which a conserved cysteine residue from the prodomain interacts with the zinc ion in the active site and prevents binding and cleavage of the substrate [5]. During maturation in the Golgi, the prodomain is cleaved by furin-like enzymes and the metalloprotease is rendered active, although other modes of ADAM activation have also been postulated [6]–[9].

ADAM12 has an active metalloprotease domain, which has been shown to cleave a range of transmembrane substrate proteins. Depending on a cellular context, substrates include members of the epidermal growth factor (EGF) family of ligands (EGF and heparin-binding-EGF) [10]–[13], the Notch pathway ligand Delta-like 1 [14], sonic hedgehog [15], receptor tyrosine kinase Tie-2 [12], vascular endothelial (VE) cadherin [12], vascular endothelial growth factor receptor 2, or Flk-1 [12], Kit ligand 1 (Kitl1) [12], Vascular cell adhesion protein 1 (VACAM-1) [12], and ephrin-A1 [16]. In addition, ADAM12 facilitates Transforming Growth Factor β (TGFβ) signaling by a mechanism that is independent of its proteolytic activity and involves the accumulation and stabilization of TGFβ type II receptor in early endosomes [17].

While ADAM12 is transiently expressed during embryonic morphogenesis of skeletal muscles, visceral organs, and bone [18], ADAM12-deficient mice do not show major developmental abnormalities [19]. Post-natal ADAM12 expression in healthy and non-injured organs is low, but it is highly elevated in diseases accompanied by fibrosis, such as liver cirrhosis [20], muscle injury [21], scleroderma [22], chronic wounds [23], and cardiac hypertrophy [24]. Consistently, a recent genetic study in mice has shown that ADAM12 is expressed in mesenchymal perivascular cells (pericytes), which are programmed during vascular wall development, are activated in response to tissue injury, and generate pro-fibrotic myofibroblasts [25]. Furthermore, ADAM12 expression is strongly elevated in many cancers, including breast, head and neck, bone, lung, bladder, prostate, and brain cancers, as well as aggressive fibromatosis [26]–[39]. Recently, ADAM12 has been shown to be involved in the formation of invadopodia, cellular structures that aid cancer cell invasion, in head and neck, lung, and pancreatic cancer cells [13]. In breast cancers, ADAM12 is selectively up-regulated in the claudin-low subtype of tumors [40], which have aggressive characteristics, molecular signatures of epithelial-to-mesenchymal transition, and are enriched in gene signatures of breast tumor-initiating cells [41]. By analyzing survival data of a large group of breast cancer patients, we have recently concluded that ADAM12 is the primary protease responsible for the activation of EGF receptor in early stage, lymph node-negative triple negative breast cancer (lacking the expression of estrogen receptor, progesterone receptor, and HER2) [42].

**Figure 1:**
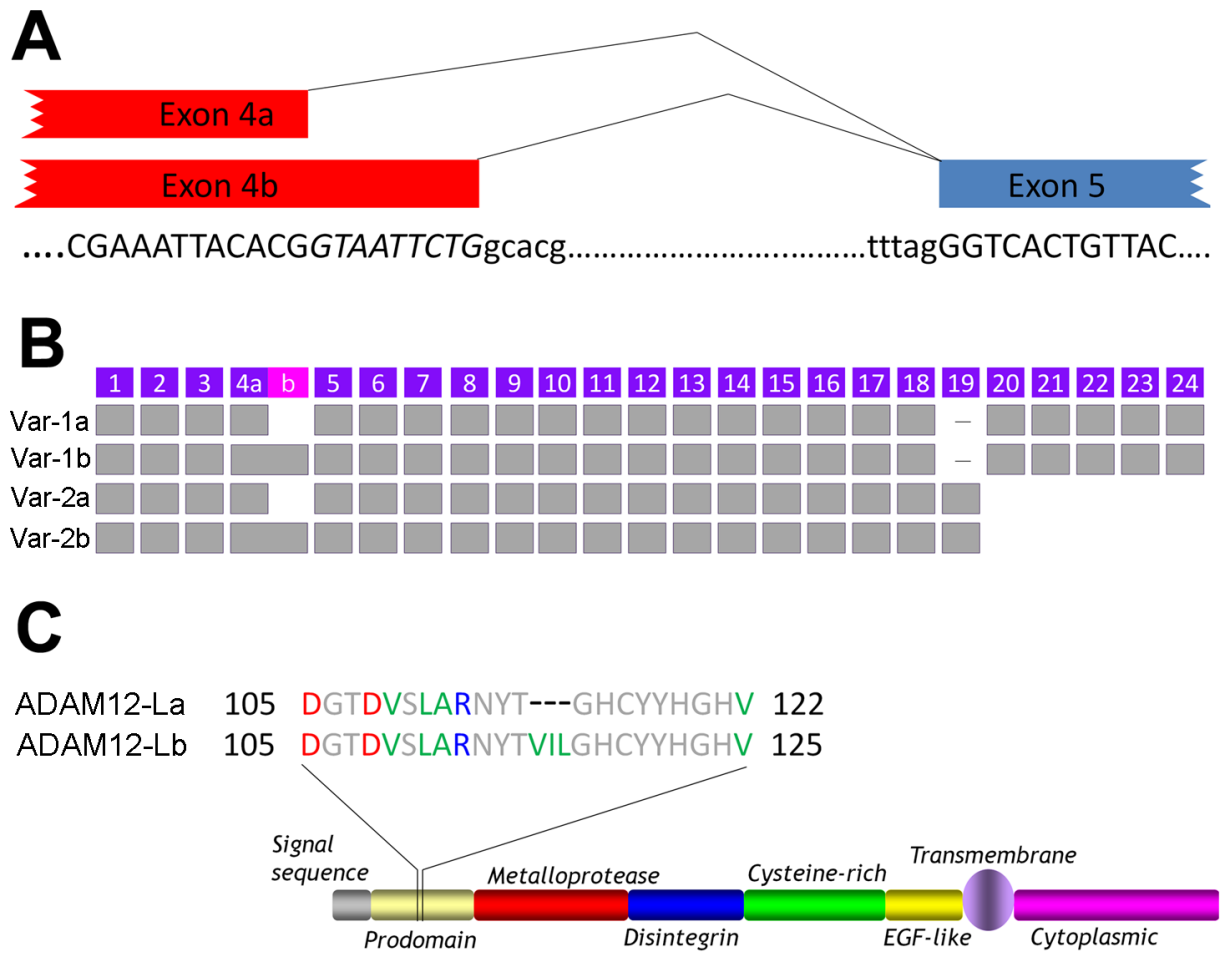
Alternative splicing of human *ADAM12* transcripts generates two ADAM12-L isoforms. (A) Diagram of the alternative mRNA splicing event at the exon 4-exon 5 junction. Capital letters represent exons and lower case letters represent the intron. The nine nucleotide sequence 5′-GTAATTCTG-3′ missing in exon 4a and present in exon 4b are shown in *italics*. (B) Exon composition of human *ADAM12* transcripts. The nine-nucleotide extension in exon 4b is shown in magenta. Exons are not drawn to scale. (C) Diagram of the two transmembrane protein isoforms: ADAM12-La, which lacks the ^114^VIL^116^ motif in the prodomain, and ADAM12-Lb, which includes this motif.

The human *ADAM12* gene is alternatively spliced, resulting in two major protein isoforms: a long, transmembrane form called ADAM12-L and a short, secreted form designated ADAM12-S [43]. The ADAM12-L isoform is encoded by transcript variant 1, or *ADAM12var-1*, which includes exons 1–18 and 20–24. ADAM12-S is encoded by transcript variant 2, or *ADAM12var-2*, which comprises exons 1–19. Furthermore, analysis of human *ADAM12* sequences present in major databases suggests that there are two forms of exon 4, with two alternative 3′ ends. The shorter form will be designated here exon 4a. The longer form, which ends 9 bp further downstream from exon 4a, will be designated exon 4b (Figure 1A). The alternative splicing event generating exon 4a and exon 4b is known as alternative 5′ splice site selection [44], [45].

**Figure 2:**
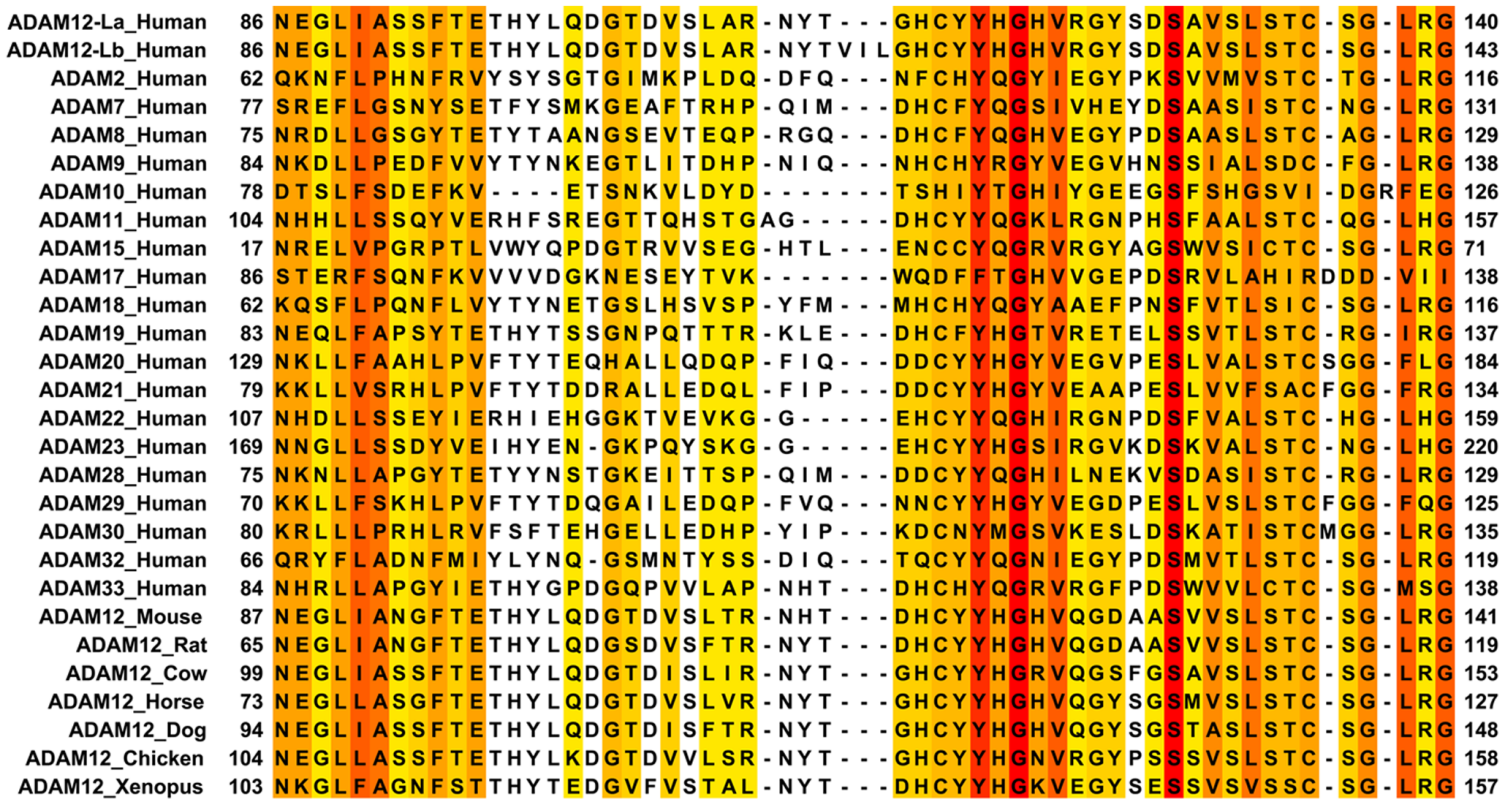
The ^114^VIL^116^ motif is not conserved between different human ADAMs and between ADAM12 from different species. Multiple sequence alignment of the region in ADAM proteins flanking the ^114^VIL^116^ motif. GenBank accession numbers are: ADAM2, NP_001455; ADAM7, NP_003808; ADAM8, NP_001100; ADAM9, NP_003807; ADAM10, NP_001101; ADAM11, NP_002381; ADAM12, NP_003465; ADAM15, AAS72997; ADAM17, NP_003174; ADAM18, NP_055052; ADAM19, NP_150377; ADAM20, NP_003805; ADAM21, NP_003804; ADAM22, NP_068369; ADAM23, NP_003803; ADAM28, NP_055080; ADAM29, NP_055084; ADAM30, NP_068566; ADAM32, NP_659441; ADAM33, NP_079496; Mouse, NP_031426; Rat, XP_001054670; Cow, NP_001001156; Horse, XP_001490097; Chicken, NP_001136322; Xenopus, NP_00035103. The dog sequence was obtained from e!Ensembl (ENSCAFP00000041414) due to the lack of a signal peptide in the GenBank sequence. Conservation strength is shown in red (high), orange (medium), yellow (poor), and white (no conservation).

Interestingly, according to the most recent releases of all major sequence databases, alternative splicing at exon 4a/b is present solely in the *ADAM12var-2* transcript, but not in the *ADAM12var-1* transcript. *ADAM12var-1b* and *ADAM12var-2b*, both containing the longer exon 4b, were first described by Gilpin et al. [46] (Figure 1B). These variants encode the ADAM12-Lb and ADAM12-Sb protein isoforms, respectively. *ADAM12var-2a*, containing the shorter exon 4a and encoding the ADAM12-Sa isoform, was later identified in a screen for novel secreted proteins [47]. *ADAM12var-1a* transcript and ADAM12-La protein isoform are not featured in any of the DNA/protein databases analyzed (Table 1). The 9-bp extension present selectively in exon 4b encodes a highly hydrophobic ^114^VIL^116^ motif in the ADAM12 prodomain (Figure 1C). This motif is not conserved in ADAM12 from other species or in other human ADAMs (Figure 2). Although the ^114^VIL^116^ sequence is not positioned in a close proximity to the known furin-like cleavage site (^204^RHKR^207^) or to the cysteine responsible for the switch mechanism (Cys179), it can potentially affect the structure and/or function of the ADAM12 protein. The high hydrophobicity of this motif might change the stability of structural motifs in the prodomain or may create a hydrophobic interface for interaction with other proteins.

**Table 1:** Accession numbers of the individual splice variants of human *ADAM12.*

	GenBank/EMBL/DDBJ		NCBI RefSeq		Ensembl		Vega		CCDS	UniProt
	mRNA	Translation	mRNA	Translation	mRNA	Translation	mRNA	Translation	mRNA and Translation	Translation
**Var-1a**	–	–	–	–	–	–	–	–	–	–
**Var-1b**	AF023476	AAC08702	NM_003474	NP_003465	ENST00000368679	ENSP00000357668	OTTHUMT00000050961	OTTHUMP00000020726	7653	O43184-1
**Var-2a**	AY358878	AAQ89237	–	–	–	–	–	–	–	O43184-3
**Var-2b**	AF023477	AAC08703	NM_021641	NP_067673	ENST00000368676	ENSP00000357665	OTTHUMT00000050962	OTTHUMP00000020727	7654	O43184-2

In this study, we sought to determine whether *ADAM12var-1a* is expressed in human cells and whether any functional differences between the ADAM12-La and ADAM12-Lb protein isoforms exist. We found that breast epithelial cells, as well as breast cancer cells, do express *ADAM12var-1a*. Most importantly, while ADAM12-La protein is proteolytically processed and trafficked to the cell surface, ADAM12-Lb is poorly processed and is retained in the ER. Furthermore, we show that the relative abundance of the endogenous ADAM12-La and ADAM12-Lb proteins detected in several breast cancer cell lines varies significantly. As the ^114^VIL^116^ motif is not conserved between species and is not found in other members of the ADAM family, we conclude that the canonical form of ADAM12-L is ADAM12-La and not ADAM12-Lb.

## Materials and Methods

### Vector Construction

Full-length human *ADAM12var-1a* cDNA was amplified from MCF10DCIS.com cells using two consecutive PCR reactions with nested primers. The first set of primers was: 5′-GGA AAT CCC TCC GGT CGC GAC-3′ (F) and 5′-ACT GAC GGC AGT AGC TCA AAG-3′ (R). The second set of primers was: 5′-TAC CTT CAA TTG TGA AGG CCG GCG ACG ATG GC-3′ (F) and 5′-ACA CGT CGA CTC ACT TAA TAT AGG CGG TGT G-3′ (R). The underlined sequences in the forward and reverse primers of the second set correspond to the beginning and the end of the coding region of human *ADAM12var-1*, respectively. The PCR product was gel-purified, digested with MfeI and SalI, and cloned into the pBABEpuro retroviral expression vector at the EcoRI and the SalI sites. The nine-nucleotide insertion 5′-GTAATTCTG-3′ in exon 4b was generated using the QuickChange Site-Directed Mutagenesis kit (Stratagene). The entire lengths of the coding regions of *ADAM12var-1a* and *ADAM12var-1b* were sequenced to confirm the presence of the insertion and to exclude any PCR errors.

### Cell Culture

MCF10DCIS.com cells were obtained from Asterand (Detroit, MI). MCF10A, MDA-MB-231, HEK293, and Hs578T cells were purchased from American Type Culture Collection (Manassas, VA). PhoenixAmpho cells, the retroviral packaging line, were obtained from Dr. Garry P. Nolan (Stanford University). MCF10DCIS.com cells were cultured in 1∶1 (v/v) Dulbecco’s Modified Eagle Medium (DMEM)/Ham’s F-12 containing 15 mM HEPES and supplemented with 5% horse serum and 29 mM sodium bicarbonate. MCF10A cells were cultured in DMEM/F-12 and supplemented with 5% horse serum, 0.5 µg/mL hydrocortisone, 20 ng/mL human EGF, 10 µg/mL insulin, 100 ng/mL cholera toxin and 1% penicillin/streptomycin. HEK293 and PhoenixAmpho cells were cultured in DMEM supplemented with 10% fetal bovine serum (FBS). Hs578T cells were cultured in DMEM containing 10% FBS and 10 µg/mL insulin. MDA-MB-231 cells were cultured in DMEM/F-12 with 10% FBS. All cells were maintained at 37°C in 5% CO_2_.

### Antibodies

Polyclonal rabbit anti-ADAM12 antibody (Ab#3394) raised against the cytoplasmic tail of human ADAM12-L [42] was used at a dilution of 1∶20,000 for Western blotting and 1∶500 for immunofluorescence. Mouse monoclonal anti-ADAM12 antibody (R&D Biosciences; clone 632525) was used for flow cytometric analysis at a dilution of 1∶100. Other antibodies were: anti-β-actin (Sigma, clone AC-15; 1∶20,000 dilution), anti-α-tubulin (Sigma, clone DM 1A; 1∶40,000 dilution), anti-KDEL, an endoplasmic reticulum marker (Enzo Life Sciences, clone 10C3; 1∶100 dilution), anti-TGN38, a trans-Golgi marker (BD Biosciences, clone 2; 1∶50 dilution), anti-EEA1, an early endosomal marker (BD Biosciences, clone 14; 1∶50 dilution), and anti-epidermal growth factor receptor (EGFR) (Cell Signaling Technologies, clone D38B1; 1∶5,000 dilution).

### Generation of Cells with Stable Overexpression of ADAM12-La or ADAM12-Lb

ADAM12-La and ADAM12-Lb were stably overexpressed in MCF10A cells using retroviral transduction. PhoenixAmpho cells were seeded in 100-mm plates 16 hours prior to transfection. pBabePuro retroviral expression vectors containing *ADAM12-var1a* or *ADAM12-var1b* sequences, or empty pBabePuro vector, were transfected into PhoenixAmpho cells using the calcium phosphate method (15 µg DNA/plate); 25 µM chloroquine was added to the medium prior to adding the DNA/CaCl_2_ solution. Cells were incubated at 37°C for 24 h, then the medium was changed to fresh DMEM +10% FBS, and cells were incubated for additional 48 h at 32°C. The medium containing retroviruses was then collected and centrifuged at 500×g for 5 min. Supernatants were supplemented with 5 µg/mL polybrene (Sigma), and were added without further dilution to MCF10A cells. Forty eight hours post-infection, cells with stable overexpression of ADAM12 proteins were selected using 2 µg/mL puromycin for 10 days.

### Knock-down of ADAM12 Expression

siRNAs specific for exon 4a and exon 4b were designed using the Thermo Scientific siDESIGN Center. The antisense strand sequence of the exon 4a specific siRNA was 5′-UAG UAA CAG UGA CCC GUG UUU-3′ and was prepared as a regular siRNA. The antisense strand sequence of the exon 4b specific siRNA was 5′-UGA CCC AGA AUU ACC GUG UUU-3′ and was prepared with the proprietary ON-TARGET modifications. The siRNAs were diluted to 20 µM in 1×siRNA buffer (Thermo Scientific) and stored at −20°C. For transfection, siRNAs were used at a final concentration of 50 nM. DharmaFECT I transfection reagent was used according to the manufacturer’s instructions. Transfection complexes were removed after 24 hours and cell lysates were collected for immunoblotting after 72 hours. In an alternative approach, ADAM12 was knocked-down using MISSION™ Lentiviral shADAM12 Transduction Particles (Sigma, clone ID TRCN0000047037), as described [40]. This shRNA clone targets the sequence GCCTGAATCGTCAATGTCAAA at the nucleotide position 1922–1942 in the ADAM12-Var1a coding sequence and 1931–1951 in the ADAM12-var1b coding sequence. Control treatment included cell incubation with MISSION™ Non-Target shRNA Control Transduction Particles (Sigma, SHC002V). Transduction was performed according to the manufacturer’s instructions. After one day, media containing lentiviral particles were replaced with fresh media, and after additional 24 h, stably transduced cells were selected with 3 µg/ml of puromycin for 7 days.

### Immunoblotting

Cells were treated with lysis buffer (50 mM Tris-HCl pH 7.4, 150 mM NaCl, 1% Triton X-100, 0.5% sodium deoxycholate, 0.1% sodium dodecylsulfate (SDS), 5 mM EDTA, 1 mM 4-(2-Aminoethyl) benzenesulfonyl fluoride hydrochloride (AEBSF), 5 µg/mL pepstatin, 5 µg/mL leupeptin, 5 µg/mL aprotinin, and 10 mM 1,10-phenanthroline). Extracts were centrifuged for 15 minutes at 21,000×g at 4°C. For overexpressed ADAM12, the supernatants were resolved using SDS-PAGE (8%) and transferred to a nitrocellulose membrane. For endogenous ADAM12 detection, supernatants were enriched for glycoproteins by binding to concanavalin A agarose (Sigma; 25 µl resin/500 µl lysate) for 2 hours at 4°C prior to SDS-PAGE. Membranes were blocked in 5% milk and 0.3% Tween-20 in Dulbecco’s Phosphate Buffered Saline (DPBS). Rabbit anti-human ADAM12 (Ab#3394), mouse anti-β-actin, mouse anti-α-tubulin, and rabbit anti-EGFR antibodies were diluted in blocking buffer and incubated with the membranes. Horseradish peroxidase-conjugated anti-rabbit or anti-mouse antibodies were used as secondary antibodies. Signal detection was performed using SuperSignal West Pico Chemiluminescent Substrate (Pierce).

### cDNA Preparation and RT-PCR

Total RNA was extracted using the Qiagen RNeasy kit. One microgram of the total RNA was treated with deoxyribonuclease I (Qiagen) and reverse-transcribed using the SuperScript III First Strand Synthesis system for RT-PCR (Invitrogen). A 100-bp fragment of ADAM12 cDNA including the exon 4-exon 5 splice junction was amplified using the following primers: 5′-GGT ACT GAT GTC TCC CTC GCT CG-3′ (F) and 5′-CGT GCT GAG ACT GAC TGC TGA ATC-3′ (R). PCR products were resolved in a 2.0% agarose/TAE gel, visualized with ethidium bromide and UV illumination, and then extracted using the Qiaex II kit (Qiagen). DNA sequencing was performed at the Kansas State University DNA Sequencing and Genotyping Facility using the Applied Biosystems 3730 DNA Analyzer. The sequence of the *ADAM12var-1a* has been deposited in GenBank under the accession number KF444157.

### Cell Surface Biotinylation

Cells grown in 6 well plates were washed with DPBS, then incubated at 4°C for 60 minutes with 2.5 mM EZ-link *N-*hydroxysuccinimide -PEG_12_-biotin (Pierce) in DPBS. Remaining free reagent was quenched using 100 mM glycine. After washing cells several times with DPBS, cell lysates were collected as described above, and a fraction was retained as the input sample. The remaining lysate was allowed to adsorb onto Neutravidin agarose resin (Pierce) for 60 minutes at 4°C. The resin was washed three times with cell lysis buffer, followed by elution with SDS sample buffer. Samples were resolved by SDS-PAGE and transferred to a nitrocellulose membrane for immunoblotting.

### Endo H Digestion

Cell lysate supernatants were prepared as described above. Supernatants were treated with 10×glycoprotein denaturation buffer (New England Biolabs) and boiled for 10 minutes. After cooling, 10×G5 buffer (New England Biolabs) was added, and each sample was divided into two parts; each part was treated with or without Endo H (New England Biolabs) at 37°C for 1 hour. The reaction was stopped by adding SDS sample buffer and boiling. Samples were resolved by SDS-PAGE and transferred to a nitrocellulose membrane for immunoblotting.

### Immunofluorescence

Cells were plated onto sterile coverslips and allowed to attach overnight. Cells were then washed and treated with 3.7% paraformaldehyde in DPBS for 20 minutes at room temperature. After the fixative was removed, cells were permeabilized with 0.1% Triton X-100 in DPBS for 5 minutes. The blocking step was performed using 1% bovine serum albumin (BSA) and 5% donkey serum in DPBS at for 30 minutes 37°C. Primary antibodies were diluted in 1% BSA in DPBS and incubation was performed for 60 minutes at 37°C. Red X-conjugated anti-rabbit (1∶200) and AlexaFluor 488-conjugated anti-mouse (1∶200) antibodies were diluted in 1% BSA in DPBS and incubated with the coverslips for 45 minutes at 37°C. After washing, coverslips were mounted onto slides and allowed to dry overnight. Slides were imaged at 63×magnification using a Zeiss Axiovert-200 inverted fluorescent microscope.

### Flow Cytometry

Cells were trypsinized into a single cell suspension, washed with DPBS and incubated with monoclonal anti-ADAM12 antibody or isotype control antibody for 30 minutes on ice. Cells were then washed 3 times, incubated with allophycocyanin (APC)-conjugated anti-mouse antibodies (Jackson ImmunoResearch; 1∶100) for 30 min on ice, and then with 1 µg/ml propidium iodide (Sigma) for viability. Analysis was performed using a BD FACSCalibur flow cytometer. Only the cells negative for propidium iodide (PI) staining (viable cells) were selected for the ADAM12 analysis.

## Results

### ADAM12 Transcripts Present in Human Mammary Epithelial Cells Contain Exon 4a and 4b

**Figure 3:**
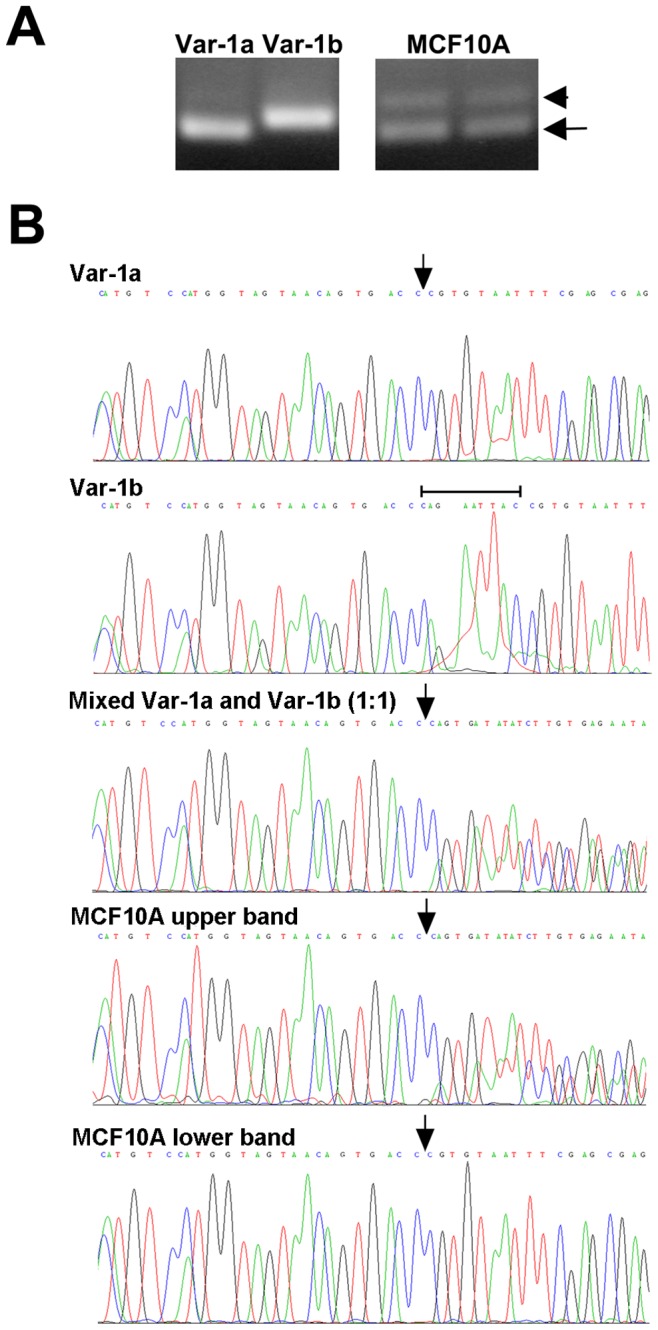
Breast epithelial cells express *ADAM12* transcripts containing exon 4a and 4b. (A) PCR amplification of a 100-bp region flanking the exon 4-exon 5 junction was performed using Var-1a and Var-1b vector controls (left) or cDNA samples from MCF10A cells. PCR products were resolved in a 2.0% agarose gel; two duplicate samples are shown for MCF10A cells. Upper and lower bands observed for MCF10A cells are indicated with arrowhead and arrow, respectively. (B) The PCR products were extracted from the gel and sequenced. The chromatograms of the sequencing reactions are shown for Var-1a and Var-1b vector controls, a 1∶1 mixture of the two vector controls, and the two PCR products (corresponding to the upper or the lower band in panel A) amplified from MCF10A cells. The bar designates the nine-nucleotide region present exclusively in exon 4b, and arrow indicates the 3′ end of exon 4a. In samples containing mixed fragments, this arrow therefore indicates the site where the sequences diverge and the chromatogram begins to show multiple peaks. Notice that the sequencing reaction was performed using a reverse primer, and the sequences shown represent reverse complements of *ADAM12* transcripts.

To determine which forms of exon 4 are present in *ADAM12* transcripts expressed by human mammary cells, we amplified a ∼100-bp region surrounding the exon 4-exon 5 splice junction using cDNA isolated from MCF10A mammary epithelial cell line. After PCR products were resolved by agarose gel electrophoresis, two bands were clearly visualized (Figure 3A). The two bands were excised from the gel and subjected to DNA sequencing. The lower band produced a single sequence that was identical to the sequence of the control vector containing a cloned *ADAM12var-1a* insert (Figure 3B). The upper band produced two sequences that had identical 5′-ends but then diverged at the site corresponding to the 3′-end of exon 4a, as indicated by the arrow. This might be due to the presence of heteroduplexes containing the 4a and 4b sequences in a non-denaturing gel, as well as unresolved 4a and 4b homoduplexes. Importantly, the chromatogram obtained for the upper band was indistinguishable from the chromatogram obtained for a mixture of *ADAM12var-1a* and *ADAM12var-1b* vector controls. We have concluded that the upper band represented, therefore, a mixture of *ADAM12* variants containing exon 4a and exon 4b.

### ADAM12-Lb is Poorly Processed and is Retained in the ER

**Figure 4:**
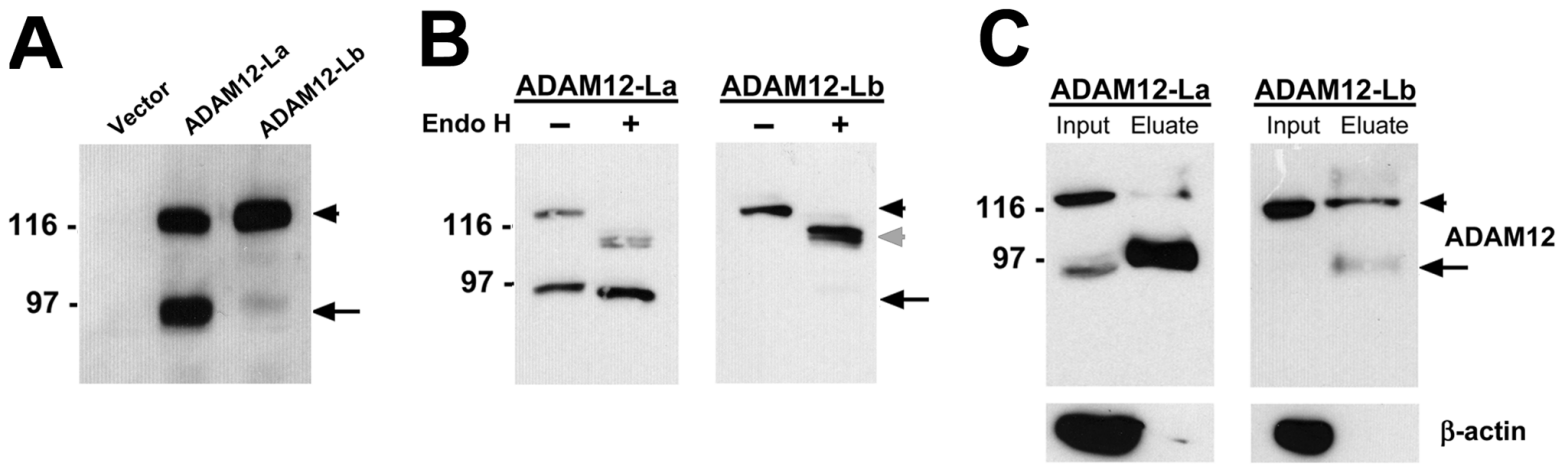
ADAM12-Lb is poorly processed and is retained in a pre-Golgi compartment. (A) Proteolytic processing of ADAM12-La and ADAM12-Lb in MCF10A cells. Cells with stable expression of ADAM12 proteins were selected with puromycin after retroviral infection. Total cell lysates were analyzed by Western blotting. The full-length protein is indicated by arrowhead, and the processed form after prodomain removal is denoted with arrow. (B) Mobility shift analysis of ADAM12-La and ADAM12-Lb after endoglycosidase H (Endo H) treatment. The nascent full-length protein and the processed form are indicated with arrowhead and arrow, respectively. The deglycosylated full-length species is denoted with grey arrow. (C) Cell surface biotinylation of MCF10A cells stably expressing ADAM12-La or ADAM12-Lb. Biotinylated proteins were isolated on Neutravidin agarose and subjected to SDS-PAGE and Western blotting. Input (1/40 of the sample volume) refers to total cell lysates prior to Neutravidin binding, and eluate (1/2 of the sample volume) refers to biotinylated proteins that bound to the resin. β-actin, an interacellular protein, is not biotinylated and does not bind to Neutravidin.

Next, we examined the effect of the ^114^VIL^116^ motif on the proteolytic processing and intracellular trafficking of ADAM12-L. The ADAM12-La and ADAM12-Lb isoforms were stably overexpressed in MCF10A cells, and cell lysates were subjected to immunoblotting using antibody specific for the intracellular domain of ADAM12-L. Cells overexpressing ADAM12-La produced two bands of similar intensities: the ∼120-kDa band representing the nascent protein, and the ∼90-kDa band corresponding to the processed form, after cleavage of the prodomain (Figure 4A). In contrast, cells overexpressing ADAM12-Lb produced a major band of ∼120 kDa, and a very weak band of ∼90 kDa (Figure 4A). A similar pattern of bands was observed when ADAM12-La and ADAM12-Lb were stably overexpressed in HEK 293 cells. Thus, while ADAM12-La was readily processed in cells, the proteolytic processing of ADAM12-Lb was much less efficient.

Since the proteolytic processing of ADAMs occurs in the Golgi apparatus, we next probed the progression of ADAM12-La and ADAM12-Lb through the secretory pathway using endoglycosidase H (Endo H). Endo H readily cleaves high mannose-type oligosaccharides but cannot remove the complex oligosaccharides that result from modification by Golgi enzymes. As expected, the ∼120-kDa form of ADAM12-La was sensitive to Endo H, and thus must have resided in the ER. The ∼90-kDa form of ADAM12-La was more resistant to Endo H and thus must have been localized to the Golgi or post-Golgi compartments (Figure 4B). Importantly, the ∼120-kDa ADAM12-Lb form was fully sensitive to Endo H, was not modified by Golgi enzymes, and thus must have been localized to a pre-Golgi compartment.

Next, we performed cell surface biotinylation of ADAM12-La and ADAM12-Lb. Live cells were incubated with an amine-reactive, membrane impermeable biotinylation reagent, biotinylated proteins were then isolated by adsorption to Neutravidin agarose beads and analyzed by Western blotting using anti-ADAM12 antibody. Comparison of the pattern of ∼120-kDa and ∼90-kDa ADAM12 bands in total cell lysates (inputs) and in eluates showed that the 90-kDa form of ADAM12-La and ADAM12-Lb was the predominant form that was biotinylated (Figure 4C). Importantly, the total amount of ADAM12-Lb protein eluted from the Neutravidin beads (the ∼120-kDa and the ∼90-kDa forms combined) was much lower than that of ADAM12-La. This result indicated that ADAM12-Lb localized to the cell surface more poorly than ADAM12-La.

**Figure 5:**
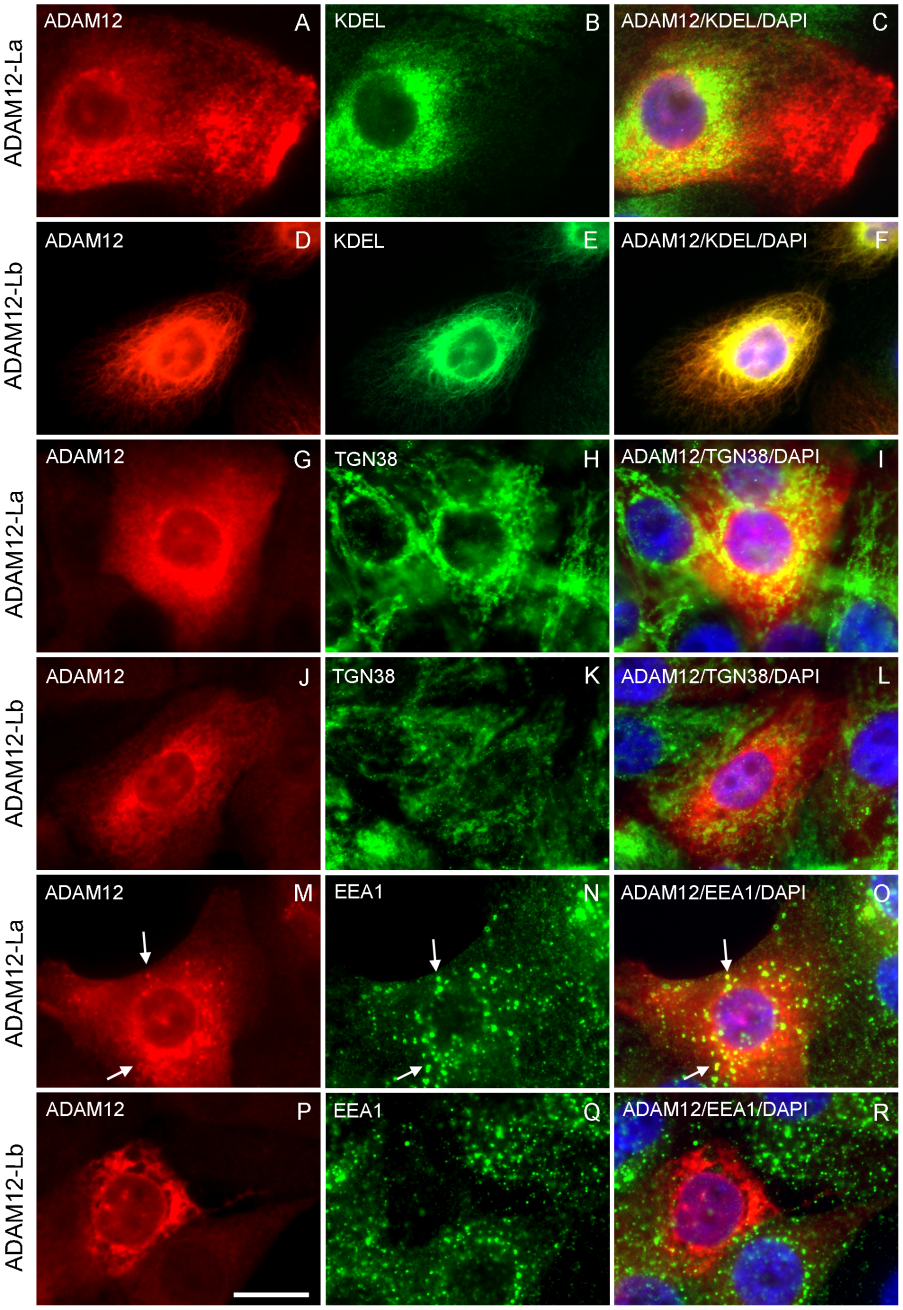
ADAM12-Lb is localized to the endoplasmic reticulum. MCF10A cells stably expressing ADAM12-La or ADAM12-Lb were fixed, permeabilized, and stained with anti- ADAM12 antibody (A, D, G, J, M, P), anti-KDEL antibody (an endoplasmic reticulum marker; B, E), anti-TGN38 antibody (a trans-Golgi marker; H, K), anti-EEA1 antibody (an early endosomal marker; N, Q), and DAPI. Overlay images are shown in panels C, F, I, L, O, R. Partial co-localization of ADAM12-La and EEA1 is indicated by white arrows. Bar, 20 µm.

To directly visualize subcellular localization of ADAM12-La and ADAM12-Lb in cells, immunofluorescence staining was performed using anti-ADAM12-L antibody and three different antibodies marking distinct compartments of the secretory pathway. Using anti-KDEL antibody, a marker of the ER, we determined that ADAM12-La only partially localized to the ER, and majority of ADAM12-La protein was found in post-ER compartments (Figure 5A–C). In contrast, anti-ADAM12 staining of ADAM12-Lb-expressing cells fully co-localized with anti-KDEL staining (Figure 5D–F). Using anti-TGN38 antibody, a *trans*-Golgi marker, we detected partial co-staining with anti-ADAM12 antibody in ADAM12-La-expressing (Fig. 5G–I), but not in ADAM12-Lb-expressing cells (Fig. 5J–L). Finally, we examined co-localization of ADAM12 with EEA1, an early endosomal marker. It was previously shown that ADAM12 is constitutively internalized from the cell surface via the clathrin-dependent pathway and is detected in both early and recycling endosomes [17], [48]. Indeed, we detected partial co-localization of ADAM12-La with EEA1 (Fig. 5M–O), but no co-localization was observed for ADAM12-Lb and EEA1 (Fig. 5P–R). In summary, these results confirmed that ADAM12-Lb is efficiently retained in the ER. To further explore whether ADAM12-Lb might have been misfolded and retained in the ER by the ER quality control system, we performed co-immunoprecipitation of ADAM12-Lb with several known ER chaperones: BiP, ERp44, ERp72, Grp94, PDI and calnexin. None of these chaperones was detected in the anti-ADAM12-Lb immunoprecipitates (results not shown). This data, together with the relative stability of the ADAM12-Lb protein, implied that the protein was not grossly misfolded.

### Breast Cancer Cell Lines Show Different Relative Expression Levels of ADAM12-La and ADAM12-Lb

**Figure 6:**
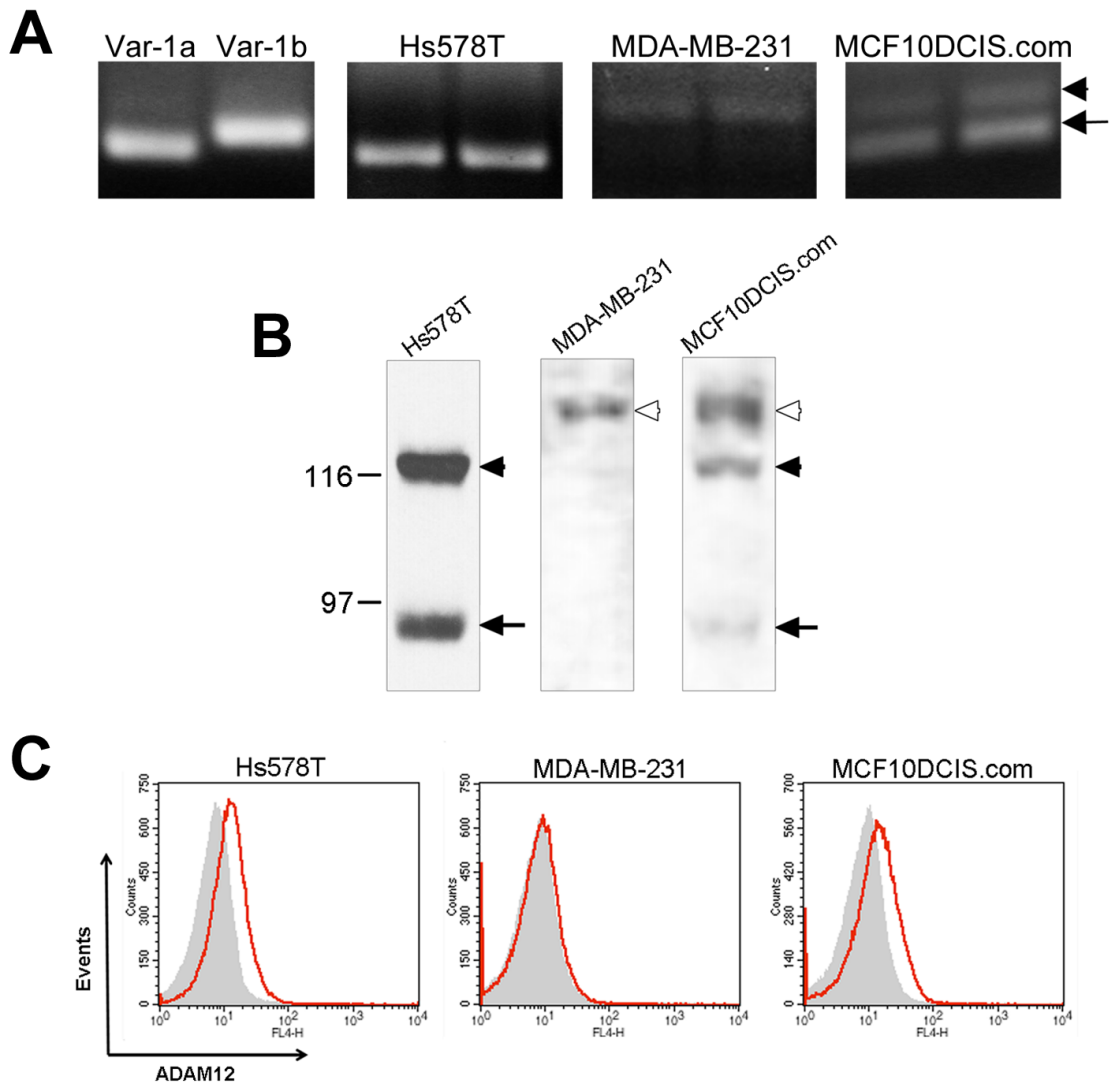
Breast cancer cell lines express different levels of ADAM12-La and ADAM12-Lb. (A) A 100-bp region flanking the exon 4-exon 5 junction was PCR-amplified using duplicate cDNA samples isolated from Hs578T, MDA-MB-231, and MCF10DCIS.com cells and analyzed as in Fig. 3. Var-1a and Var-1b vectors served as controls. Exon 4a- and exon 4b-related bands are indicated with arrow and arrowhead, respectively. (B) Glycoprotein-enriched fractions from Hs578T, MDA-MB-231, and MCF10DCIS.com cells were analyzed by Western blotting using anti-ADAM12-L antibody. Full length ADAM12-La and ADAM12-Lb are indicated with solid and open arrowheads, respectively. The processed form of ADAM12-La is shown with arrow. (C) Cell surface localization of ADAM12-L was examined by flow cytometry. Live cells were trypsinized and stained with an antibody specific for the extracellular domain of ADAM12-L (red) or with isotype control antibody (grey).

Next, we analyzed ADAM12-La and ADAM12-Lb expression in three cancer cell lines, Hs578T, MDA-MB-231, and MCF10DCIS.com. Hs578T and MDA-MB-231 cell lines represent claudin-low breast cancer cell lines, in which high levels of *ADAM12* transcript variant 1 were detected by microarray profiling [41]. MCF10DCIS.com is a breast cancer cell line derived from a tumor originating from xenografting premalignant MCF10AT cells into severe combined immunodeficient mice [49], [50]. MCF10DCIS.com cells are frequently used to model early breast cancer, and they express high levels of *ADAM12* transcript variant 1 [40]. First, we amplified a ∼100-bp region surrounding the exon 4-exon 5 splice junction in *ADAM12* transcripts using cDNA isolated from Hs578T, MDA-MB-231, and MCF10DCIS.com cells and the same primer set as in Figure 3 above. The PCR band pattern was different for each of these three cell lines, with a single band indicative of exon 4a present in Hs578T cells, a predominant band indicative of exon 4b in MDA-MB-231 cells, and two bands detected in MCF10DCIS.com cells (Figure 6A). Consistently, Western blotting showed that Hs578T cells express high levels of ADAM12-La, whereas ADAM12-Lb is undetected in these cells (Figure 6B). Conversely, MDA-MB-231 express high levels of ADAM12-Lb, while ADAM12-La is below the detection limit. MCF10DCIS.com cells express both ADAM12-La and ADAM12-Lb isoforms. The apparent molecular weight of ADAM12-Lb in MDA-MB-231 and MCF10DCIS.com cells (∼125-kDa) was noticeably larger than the molecular weight of the full-length ADAM12-La (∼120-kDa) despite only a three-amino acid difference (^114^VIL^116^) in their prodomains, which might be due to different glycosylation or other post-translational modifications. Analysis of live cells by flow cytometry using an antibody specific for the extracellular domain of ADAM12-L demonstrated that Hs578T and MCF10DCIS.com cells both had detectable levels of ADAM12-L at the surface, while MDA-MB-231 did not (Figure 6C). Additional immunoprecipitation experiments confirmed that this antibody recognized ADAM12-La and ADAM12-Lb equally well (Figure S1). Therefore, the lack of detection of ADAM12-L at the surface of live MDA-MB-231 cells corroborates the notion that these cells express predominantly the ADAM12-Lb form that is retained in the ER and not proteolytically processed in the Golgi. In contrast, Hs578T and MCF10DCIS.com cells express sizeable amounts of ADAM12-La that is transported out of the ER, is proteolytically processed in the Golgi, and is trafficked to the cell surface.

**Figure 7:**
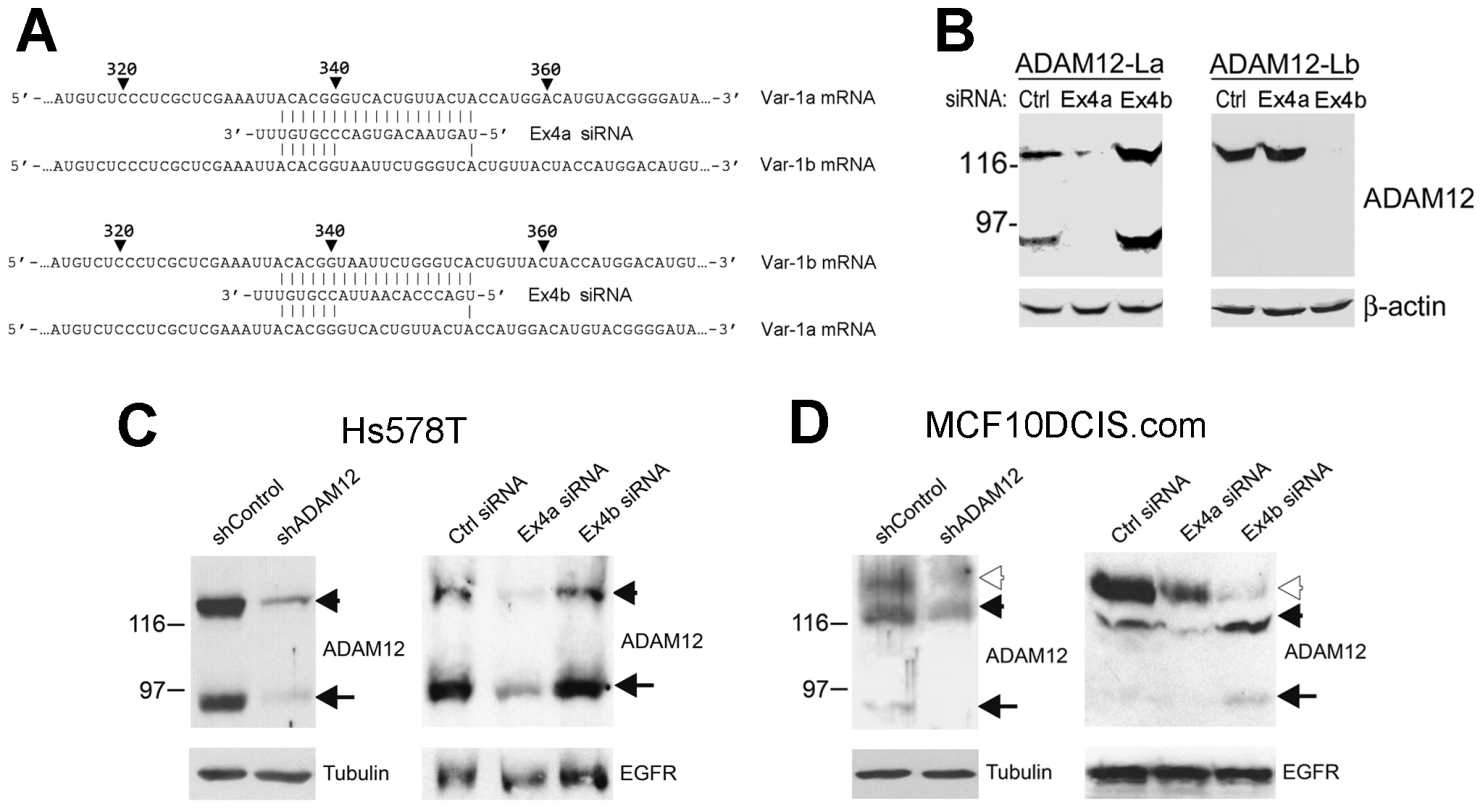
Confirmation of the identities of the endogenous ADAM12-La and ADAM12-Lb proteins detected in breast cancer cell lines. (A) Sequences of two siRNAs designed to specifically target *ADAM12* exon 4a (Ex4a) or exon 4b (Ex4b). The siRNA antisense strands are shown, and possible base pairing between each siRNA and Var-1a or Var-1b mRNA is indicated. The numbers refer to the nucleotide position in the coding sequence for each mRNA variant. (B) Efficacies and specificities of the two siRNAs. HEK293 cells stably expressing ADAM12-La or ADAM12-Lb were transfected with negative control siRNA (Ctrl), Ex4a siRNA, or Ex4b siRNA. Total cell lysates were analyzed by Western blotting using anti-ADAM12 antibody. β-actin is a loading control. (C,D) ADAM12-L knockdown in Hs578T and MCF10DCIS.com cells. Cells were stably transduced with lentiviruses bearing an shRNA construct targeting both *ADAM12var-1a* and *ADAM12var-1b* or control viruses. Alternatively, cells were transiently transfected with Ex4a siRNA, Ex4b siRNA, or negative control siRNA. Total cell lysates in C and D were enriched for glycoproteins on concanavalin A agarose prior to analysis. Full length ADAM12-La and ADAM12-Lb are indicated with solid and open arrowheads, respectively. The processed form of ADAM12-La is indicated with arrow. Epidermal Growth Factor Receptor (EGFR), ß-actin, and tubulin are gel-loading controls.

To further confirm the identity of the ADAM12-La and ADAM12-Lb proteins detected in cancer cells, we generated siRNAs selectively targeting each variant (Figure 7A). The efficacy and specificity of these siRNAs were tested in HEK293 cells stably overexpressing ADAM12-La or ADAM12-Lb. The exon 4a siRNA efficiently knocked down ADAM12-La and had much lesser effect on ADAM12-Lb (Figure 7B). The exon 4b siRNA potently knocked down ADAM12-Lb and, unexpectedly, increased the level of ADAM12-La (Figure 7B). Although the mechanism of this increased expression of ADAM12-La by exon 4b siRNA is not clear, we have concluded that both siRNAs efficiently down-regulated the cognate ADAM12-L isoforms and thus were suitable for distinguishing between the endogenous ADAM12-La and ADAM12-Lb isoforms.

We selected Hs578T and MCF10DCIS.com cells for the siRNA analysis, because these cells expressed significantly more ADAM12-L than MDA-MB-231 cells. As expected, the two forms of ∼120 kDa and ∼90 kDa observed in Hs578T cells were down-regulated by an shRNA construct targeting both ADAM12-La and ADAM12-Lb (Figure 7C). When Hs578T cells were transfected with exon 4a-specific siRNA, both ∼120-kDa and ∼90-kDa isoforms were also reduced. In contrast, exon 4b siRNA did not down-regulate the ∼120-kDa or the ∼90-kDa form in Hs578T cells (Figure 7C). Thus, ADAM12-La represents the major ADAM12-L form in Hs578T cells. In MCF10DCIS.com cells, all three ADAM12-L forms of ∼125 kDa, 120 kDa, and ∼90 kDa were reduced by an shRNA construct targeting sequences common for both ADAM12-La and ADAM12-Lb (Figure 7D). Furthermore, exon 4a siRNA primarily diminished the ∼120-kDa and ∼90-kDa forms, and exon 4b diminished the ∼125-kDa form (Figure 7D), indicating that these cells express both the ADAM12-La and ADAM12-Lb isoforms. Collectively, the results presented in Figures 6 and 7 suggest that both ADAM12-La and ADAM12-Lb are expressed at the endogenous levels in breast cancer cells, but their relative contribution to the overall ADAM12-L expression varies.

## Discussion

In this work, we show that two alternative versions of exon 4 in human *ADAM12* transcripts exist, namely exon 4a and 4b. They arise as a result of alternative 5′ (or donor) splice site usage in intron 4–5. The two alternative donor sites are located 9 nt apart and are an example of tandem sites [45]. Both versions of exon 4 can be detected in *ADAM12* transcripts present in cultured human cells. Remarkably, *ADAM12* transcript variant 1 currently featured in all publicly available databases contains exclusively exon 4b. *ADAM12* transcript variant 1 with exon 4a, *ADAM12var-1a*, is not included in any of these databases. The translation products of variant 1 comprising exon 4a or 4b, ADAM12-La and ADAM12-Lb, differ by the sequence ^114^VIL^116^ in the prodomain, which is present only in the ADAM12-Lb protein isoform. Since the amino acid sequences of ADAM12 from other species, as well as sequences of other ADAMs, do not contain the VIL motif in their prodomains, we believe that the ADAM12-La protein isoform (and the corresponding *ADAM12* transcript variant 1a) should be considered the “canonical” form of ADAM12.

We show that the ^114^VIL^116^ motif has a dramatic impact on the intracellular trafficking and proteolytic processing of ADAM12-L protein. Unlike ADAM12-La, ADAM12-Lb protein containing this motif is retained in the ER and is not transported to the cell surface. The mechanisms responsible for the retention of ADAM12-Lb in the ER are not known. ADAM12-Lb does not seem to be grossly misfolded because: (a) it does not interact with major ER chaperones, (b) it is glycosylated, and (c) it is not rapidly degraded by the ER-associated protein degradation (ERAD) [51]. The question then arises: Is ADAM12-Lb catalytically active? According to the commonly accepted view of ADAM activation, proteolytic removal of the prodomain in the Golgi is required for the disruption of the interaction between the inhibitory cysteine in the prodomain and the zinc ion in the active site. Since ADAM12-Lb contains an intact prodomain, it is expected to be catalytically inactive. However, according to other unconventional models of ADAM activation, conformational changes or protein-protein interactions involving the prodomain may be sufficient to disrupt the cysteine-zinc interaction. 69. In such case, ADAM12-Lb may be, at least partially, catalytically active. Direct detection of ADAM12-Lb activity might prove challenging, however, since no potential ADAM12 substrates have been identified in the ER so far. Although we did not study the proteolytic processing and trafficking of ADAM12-S here, we predict that the ^114^VIL^116^ motif exerts a similar effect on ADAM12-S as it does on ADAM12-L, i.e., ADAM12-Sa should be processed and secreted, whereas ADAM12-Sb is most likely retained in the ER and is not processed.

The mechanisms dictating the selection of two alternative donor splice sites in intron 4–5 of the *ADAM12* transcripts are not clear. According to the Splice-Site Analyzer Tool (http://ibis.tau.ac.il/ssat/SpliceSiteFrame.htm, Ref. [52]), the splice site score for the sequence ACG/GTAATT at the donor site of exon 4a is 78.51, for the sequence CTG/GCACTG at the donor site of exon 4b this score is 63.57, and the score for a perfect donor site sequence CAG/GTAAGT is 100 (“/” indicate the exon/intron junction). Thus, the strength of the donor site in exon 4a seems somewhat higher that that in exon 4b. However, apart from the strength of donor sites, splice site selection is regulated by *cis*-acting elements such as exonic splicing enhancers (ESEs) or silencers (ESSs), and intronic splicing enhancers (ISEs) or silencers (ISSs). These splicing regulatory elements (SREs) recruit *trans*-acting splicing factors such as serine-arginine-rich (SR) proteins or heterogeneous nuclear ribonucleoprotein (hnRNPs), which activate or suppress splice site recognition [44], [45]. Indeed, multiple SREs are detected in exon 4 a/b and intron 4–5 by the ESR Research (http://esrsearch.tau.ac.il/) and ACESCAN2 (http://genes.mit.edu/acescan2/) site prediction tools (see also below).

An important question is whether the ratio of splicing at the two tandem sites, eventually translating into the ratio of ADAM12-La and ADAM12-Lb proteins, is maintained at a fixed level or whether it varies. Our examination of three different breast cancer cell lines indicates that the relative abundance of ADAM12-La and ADAM12-Lb is different in these lines. Interestingly, two of these cell lines, Hs578T and MDA-MB-231, have been classified as claudin-low, and all three express high levels of *ADAM12* transcript variant 1 mRNA (*ADAM12var-1a* and*/*or *ADAM12var-1b*), as determined by microarray profiling [41]. However, the predominant protein isoform in Hs578T cells is ADAM12-La, while MDA-MB-231 cells seem to express mainly the ADAM12-Lb protein isoform. MCF10DCIS.com cells, in contrast, express both ADAM12-La and ADAM12-Lb forms at a comparable level. Thus, individual genetic variations such as single nucleotide polymorphisms (SNPs) may contribute to the differences in the relative abundance of *ADAM12var-1a*/ADAM12-La and *ADAM12var-1b*/ADAM12-Lb.

It has been shown that naturally occurring polymorphisms can lead to the disappearance of a selective splice variant [53]. The donor splice site of exon 4b comprises an intronic SNP C>T at chr10∶127,843,783 (variation name: rs201532694). However, the splice site score for the sequence containing this SNP, CTG/GCATTG, is 64.36, and thus it is similar to the score of the main variant CTG/GCACTG (see above). There are five other intronic SNPs positioned close to the 5′ end of intron 4–5 and three synonymous SNPs in exon 4a (http://uswest.ensembl.org/Homo_sapiens/). Interestingly, these SNPs are located within the predicted SREs and thus they may cause variations in alternative splicing at the exon 4a/4b tandem sites.

Alternative splicing has been previously described for a number of *ADAM* genes. The most common splicing events among *ADAMs* include the inclusion or skipping of entire exons. The alternative use of cytosolic-encoding exons in human *ADAM15* generates ADAM15 protein isoforms with differential propensities to regulatory cytosolic interactions [54], [55]. Interestingly, aberrant patterns of *ADAM15* splice variants have been observed in human breast cancer cells [56]. Tissue specific insertion or skipping of cytosolic-encoding exons has been also reported for *ADAM22* [57], [58]. Furthermore, alternative usage of exons encoding membrane-proximal regions in the extracellular domains of ADAM9, 12, 19, 28, and 33 gives rise to circulating forms of the proteins, which lack the membrane-spanning and cytoplasmic domains [8], [37], [59]–[64]. Our studies provide for the first time an evidence of alternative 5′ splice site selection in an *ADAM* mRNA that results in variations in the amino acid sequence of the prodomain, altered intracellular protein trafficking, and most likely an altered enzyme functionality.

## Supporting Information

Figure S1 **The anti-ADAM12 antibody (R&D Biosciences; clone 632525) used for flow cytometry recognizes both ADAM12-La and ADAM12-Lb.** MCF10A cells stably transduced to express ADAM12-La or ADAM12-Lb were subjected to immunoprecipitation using mouse anti-ADAM12 antibody, clone 632525, and Protein G agarose. IgG isotype control was used as a negative control. Equal volumes of total cell lysates of ADAM12-La- and ADAM-Lb-expressing cells (Inputs), as well as equal volumes of Protein G eluates, were analyzed by Western blotting using rabbit polyclonal anti-ADAM12 antibody #3394.
(PDF)
