# Supplementary figures and images for: Alternative mRNA Splicing Generates Two Distinct ADAM12 Prodomain Variants
Source: PLoS One. 2013 Oct 7;8(10):e75730. doi: 10.1371/journal.pone.0075730 (PMC3792144; doi:10.1371/journal.pone.0075730)

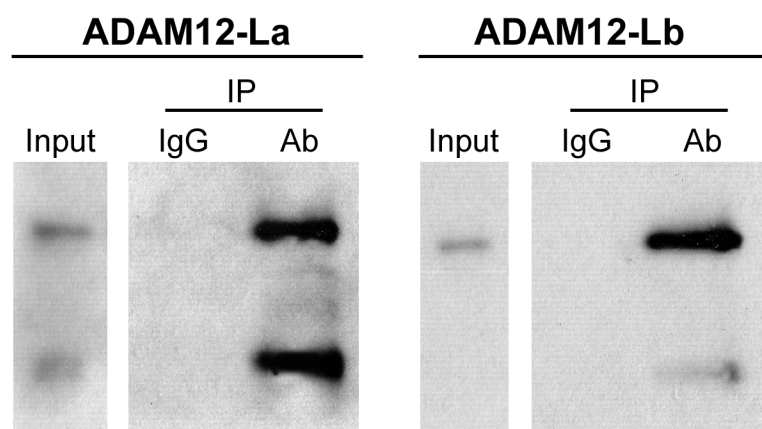

Supplement: Figure S1 — The anti-ADAM12 antibody (R&D Biosciences; clone 632525) used for flow cytometry recognizes both ADAM12-La and ADAM12-Lb. MCF10A cells stably transduced to express ADAM12-La or ADAM12-Lb were subjected to immunoprecipitation using mouse anti-ADAM12 antibody, clone 632525, and Protein G agarose. IgG isotype control was used as a negative control. Equal volumes of total cell lysates of ADAM12-La- and ADAM-Lb-expressing cells (Inputs), as well as equal volumes of Protein G eluates, were analyzed by Western blotting using rabbit polyclonal anti-ADAM12 antibody #3394. (PDF) [file pone.0075730.s001.pdf]
